# The NifZ accessory protein has an equivalent function in maturation of both nitrogenase MoFe protein P-clusters

**DOI:** 10.1074/jbc.RA119.007905

**Published:** 2019-03-07

**Authors:** Emilio Jimenez-Vicente, Zhi-Yong Yang, Julia S. Martin del Campo, Valerie L. Cash, Lance C. Seefeldt, Dennis R. Dean

**Affiliations:** From the ‡ Department of Biochemistry, Virginia Polytechnic Institute, Blacksburg, Virginia 24061 and; the § Department of Chemistry and Biochemistry, Utah State University, Logan, Utah 84322

**Keywords:** nitrogenase, nitrogen fixation, metalloenzyme, nitrogen metabolism, iron-sulfur protein, Azotobacter vinelandii, FeMo-cofactor, NifW, NifZ, P-cluster, maturation

## Abstract

The Mo-dependent nitrogenase comprises two interacting components called the Fe protein and the MoFe protein. The MoFe protein is an α_2_β_2_ heterotetramer that harbors two types of complex metalloclusters, both of which are necessary for N_2_ reduction. One type is a 7Fe-9S-Mo-C-homocitrate species designated FeMo-cofactor, which provides the N_2_-binding catalytic site, and the other is an 8Fe-7S species designated the P-cluster, involved in mediating intercomponent electron transfer to FeMo-cofactor. The MoFe protein's catalytic partner, Fe protein, is also required for both FeMo-cofactor formation and the conversion of an immature form of P-clusters to the mature species. This latter process involves several assembly factors, NafH, NifW, and NifZ, and precedes FeMo-cofactor insertion. Here, using various protein affinity–based purification methods as well as *in vivo*, EPR spectroscopy, and MALDI measurements, we show that several MoFe protein species accumulate in a NifZ-deficient background of the nitrogen-fixing microbe *Azotobacter vinelandii*. These included fully active MoFe protein replete with FeMo-cofactor and mature P-cluster, inactive MoFe protein having no FeMo-cofactor and only immature P-cluster, and partially active MoFe protein having one αβ-unit with a FeMo-cofactor and mature P-cluster and the other αβ-unit with no FeMo-cofactor and immature P-cluster. Also, NifW could associate with MoFe protein having immature P-clusters and became dissociated upon P-cluster maturation. Furthermore, both P-clusters could mature *in vitro* without NifZ. These findings indicate that NifZ has an equivalent, although not essential, function in the maturation of both P-clusters contained within the MoFe protein.

## Introduction

Efforts to transfer an efficient ability to fix dinitrogen by microbial diazotrophs to eukaryotic organisms critically depend on understanding how the catalytic components of biological nitrogen fixation are assembled. In the present work, the role of NifZ in the maturation of the MoFe protein component of nitrogenase is explored.

**Figure 1.**
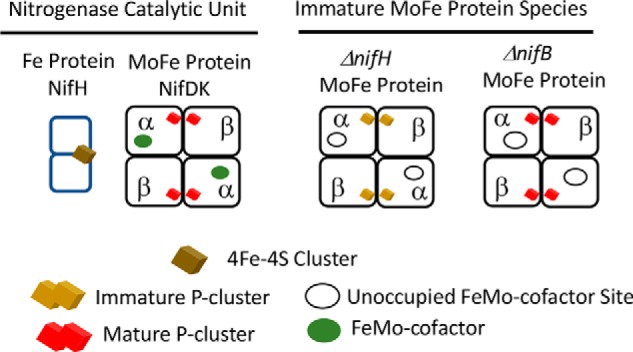
***Schematic representation* of WT molybdenum nitrogenase components and immature MoFe protein produced in Δ*nifH* and Δ*nifB* backgrounds.** For simplicity, accessory proteins associated with immature forms of MoFe protein produced in NifB- or NifH-deficient backgrounds are not indicated.

Mo-dependent nitrogenase catalyzes the reduction of inert dinitrogen gas (N_2_) and comprises two interacting components called the Fe protein and the MoFe protein (Fig. 1). The MoFe protein is an α_2_β_2_ heterotetramer that harbors two types of complex iron- and sulfur-containing metalloclusters, both of which are necessary to support N_2_ reduction (1, 2). One type is a 7Fe-9S-Mo-C-homocitrate species designated FeMo-cofactor that provides the N_2_-binding catalytic site. The other is an 8Fe-7S species designated the P-cluster, which is involved in mediating intercomponent electron transfer to FeMo-cofactor (3–6). Because the MoFe protein is a heterotetramer, it carries two pairs of its associated metal-containing cofactors that constitute two catalytic units involving one P-cluster and one FeMo-cofactor. Fe protein, the MoFe protein's catalytic partner, participates in a cyclic process involving association and dissociation with the MoFe protein, intercomponent electron transfer, and nucleotide hydrolysis. In addition to its role in catalysis, the Fe protein is required for FeMo-cofactor formation (7) and for P-cluster maturation (8). Namely, inactivation of the Fe protein results in formation of an inactive MoFe protein that contains no FeMo-cofactor as well as an Fe-S–containing P-cluster precursor of unknown structure (7, 9). FeMo-cofactor is separately synthesized (10, 11), and its insertion into FeMo-cofactorless MoFe protein is believed to occur only after P-cluster maturation is complete (8, 12). A *schematic representation* of MoFe protein and its associated metalloclusters as well as MoFe proteins produced by certain strains deficient in metallocluster assembly are depicted in Fig. 1.

**Figure 2.**
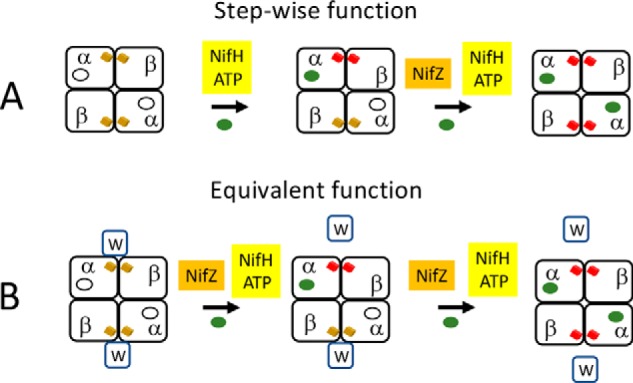
**Comparison of the “stepwise function” (*A*) and “equivalent function” (*B*) models for the participation of NifZ in P-cluster maturation.** In the stepwise function model, NifZ is not required for maturation of the “first” P-cluster but is required for the maturation of the “second” P-cluster. In the equivalent function model, NifZ participates in the formation of both P-clusters but is not required for maturation of either one. The equivalent function model also incorporates the finding in the present work that one NifW can bind to each MoFe protein αβ-unit harboring an immature P-cluster and dissociates upon P-cluster maturation.

A variety of assembly factors are either required for or assist in the formation of the metalloclusters necessary for nitrogenase activity (10, 11, 13). For example, NifH (Fe protein), NifU, NifS, NifB, NifEN, and NifV represent the minimum set of proteins required for *in vivo* formation of FeMo-cofactor, which is separately synthesized and then inserted into an apo-form of the MoFe protein already containing intact P-clusters (Fig. 1) (14, 15). Although details of P-cluster formation are not yet known, three accessory proteins, NafH, NifW, and NifZ, as well as NifH have been shown to sequentially and differentially interact with MoFe protein during the process of P-cluster formation/maturation (13). Inactivation of NifZ, which functions after NifW, produces MoFe protein containing some FeMo-cofactor, as well as a mixture of both mature and immature P-clusters. The prevailing model for NifZ involvement in P-cluster maturation (8, 16–18) is that maturation of “one” of the P-clusters requires only Fe protein, whereas maturation of the “other” P-cluster requires both Fe protein and NifZ (Fig. 2*A*). The foundational basis for this “stepwise function” model is the claim that isolated MoFe protein produced by a strain deleted for *nifZ* exhibits ∼50% of activity compared with MoFe protein produced by a WT strain and that the MoFe protein species produced by a strain deleted for *nifZ* is homogeneous (16). The suggestion that NifZ is involved in the maturation of only one half of the symmetrical α_2_β_2_ MoFe protein is not only counterintuitive; it does not adequately explain the originally reported phenotype (19) of the *nifZ*-deletion strain from the model nitrogen-fixing microbe *Azotobacter vinelandii* used for these studies. Namely, upon a shift from growth medium replete with a fixed nitrogen source, such as urea, to a nitrogen-deficient growth medium, a process usually referred to as “nitrogenase derepression,” WT *A. vinelandii* is able to continue growth as a consequence of the rapid accumulation of fully active nitrogenase components. In contrast, under the same derepression conditions, a strain deleted for *nifZ* exhibits a significant lag in growth (19). If NifZ activity is only required for maturation of one half of the MoFe protein, no such lag would be expected. Instead, assuming that nitrogen fixation is growth rate–limiting under these conditions, immediate growth for the *nifZ*-deleted strain would be predicted, albeit at half the rate of the WT strain. Furthermore, MoFe protein measured in extracts of the *nifZ*-deletion strain following a 3 h shift to nitrogen-deficient conditions exhibited only ∼30% of the MoFe protein activity produced by the WT strain, not 50% (19). These observations led us to consider a different model (Fig. 2*B*) for the involvement of NifZ in P-cluster maturation wherein NifZ has an equivalent function in the maturation of both P clusters. In this “equivalent function” model, NifZ, together with NifH, is involved in the maturation of both P-clusters contained within each α_2_β_2_ MoFe protein but is not essential for maturation of either one. Namely, the loss of NifZ slows the maturation of both P-clusters but does not prevent the *in vivo* maturation of either one. In the present work, both the stepwise function and equivalent function models for P-cluster maturation were tested by asking whether different MoFe protein species representing different stages in P-cluster maturation are produced by *nifZ*-deficient strains.

## Results

### Strains and MoFe protein designations

Strains used in the present work are listed in Table 1. Each strain carries either a polyhistidine-encoding tag or a Strep-encoding tag incorporated into the N-terminal coding region of the MoFe protein α-subunit to aid purification by application of affinity purification procedures (20, 21). Neither tag significantly impairs MoFe protein activity (13, 20, 21). In this work, affinity-tagged MoFe proteins are indicated by a superscript to indicate the particular tag for that protein (MoFe protein^His^ or MoFe protein^Str^), and MoFe proteins produced in assembly-deficient backgrounds are indicated by an additional superscript indicating the deficient gene; for example, MoFe protein^HisΔZ^ and MoFe protein^StrΔZ^ carry the corresponding affinity tag and are deleted for *nifZ*. The strain producing MoFe protein^HisΔZ^ (DJ1182) is identical to the strain used in prior work (16) leading to the stepwise function model and was produced from a parental strain (DJ1141; Table 1) and plasmid (pDB264) constructed in this laboratory. Consequently, no differences in work reported here and that previously reported (8, 16, 17, 22) can be attributed to strain differences.

**Table 1:** **Strains used in this study**

Strain	Genotype
DJ0033	Δ*nifDK*
DJ1141	MoFe protein^His^
DJ1182	MoFe protein^HisΔZ^
DJ2102	MoFe protein^Str^
DJ2106	MoFe protein^StrΔH^
DJ2107	MoFe protein^StrΔB^
DJ2111	MoFe protein^StrΔZ^
DJ2122	MoFe protein^StrΔZΔH^

### EPR spectra of MoFe protein species produced from different genetic backgrounds

**Figure 3.**
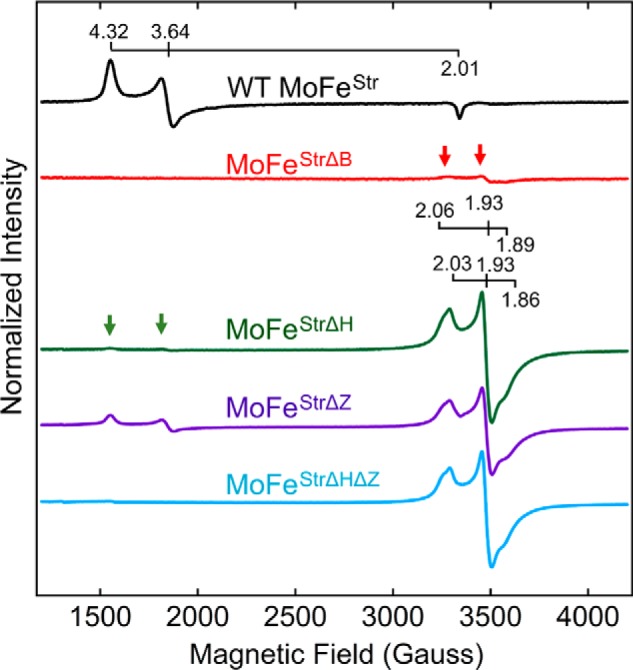
**X-band EPR spectra of resting state Strep-tagged MoFe proteins purified from different *A. vinelandii* strains.** All samples are Na_2_S_2_O_4_-reduced. EPR conditions are described in detail under “Experimental procedures.” The *S* = 32 EPR signature characteristic of FeMo-cofactor (g = 4.32, 3.64, 2.01) and the *S* = ½ signature associated with immature P-cluster appearing as two electronic isomers having similar g-values (g = 2.03, 1.93, and 1.86 and g = 2.06, 1.93, and 1.89) are indicated. All spectra were normalized to a final MoFe protein concentration of 43.5 μm. A minor immature P-cluster species, indicated by *red arrows*, is evident in the MoFe protein^StrΔB^ spectrum, and a minor FeMo-cofactor species, indicated by *green arrows*, is evident in the MoFe protein^StrΔH^ spectrum. The origin of these signals has been reported previously (9, 13, 20).

The perpendicular mode EPR spectra of WT MoFe protein^Str^, as well as MoFe protein^Str^ produced in various genetic backgrounds impaired in the maturation process, are shown in Fig. 3. These spectra illustrate aspects of various MoFe proteins produced in different genetic backgrounds and provide a basis for the interpretation of experimental results to follow. WT MoFe protein containing intact FeMo-cofactor and intact P-cluster exhibits an *S* = 32 EPR signature characteristic of FeMo-cofactor (g = 4.32, 3.64, 2.01). Intact P-cluster contained in mature MoFe protein^Str^ does not exhibit a signature in perpendicular mode EPR (Fig. 3, *black trace*) (13, 20). NifB is required for FeMo-cofactor formation but not for P-cluster maturation (13, 20). Therefore, MoFe protein^StrΔB^, which contains no FeMo-cofactor but does contain intact P-clusters, exhibits no significant EPR spectrum, except for a trace level of *S* = ½ signal attributed to P-cluster precursor (Fig. 3, *red trace*; also see comment in the Fig. 3 legend), (13, 20). MoFe protein^StrΔH^ exhibits only a trace level *S* = 32 signal but does exhibit a prominent *S* = ½ signal characteristic of the immature P-cluster species (Fig. 3, *green trace*) (8, 13). These features are in line with a requirement of Fe protein, encoded by *nifH*, to produce FeMo-cofactor (7, 23) as well as its involvement in the maturation of P-cluster precursor (8, 13). The *S* = ½ signature associated with immature P-cluster appears as two electronic isomers having similar g-values (g = 2.03, 1.93, and 1.86 and g = 2.06, 1.93, and 1.89). It has been previously shown that the very low level of *S* = 32 signal associated with MoFe protein^StrΔH^, noted in Fig. 3 (*green trace*), arises from a very weak capacity of the Fe protein associated with the V-dependent nitrogenase, product of *vnfH*, to support FeMo-cofactor formation (13, 24). MoFe protein^StrΔZ^ exhibits a small amount of the *S* = 32 signature characteristic of FeMo-cofactor as well as the *S* = ½ signature of P-cluster precursor (Fig. 3, *purple trace*). How both of these signals can be present in such samples has been rationalized by the stepwise function model shown in Fig. 2*A* (8, 16, 17) and was further explored in experiments described below. MoFe protein^StrΔZΔH^ exhibits only the *S* = ½ signature of immature P-cluster (Fig. 3, *blue trace*), further indicating the involvement of Fe protein in FeMo-cofactor formation and P-cluster maturation.

### Phenotypic characterization of nifZ-deletion strains

**Figure 4.**
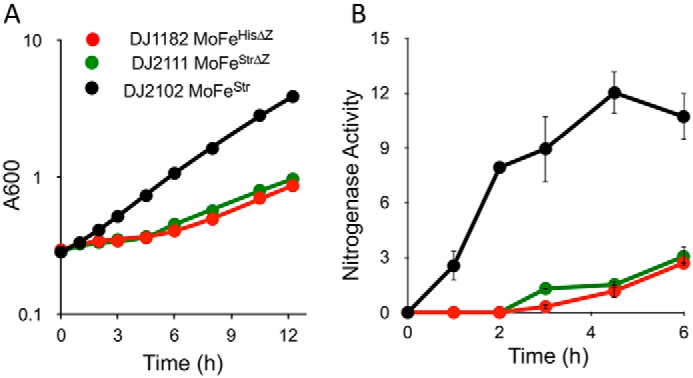
**Growth and *in vivo* nitrogenase activities.** *A*, growth of *A. vinelandii* WT (DJ2102) and Δ*nifZ* mutants (DJ2111 and DJ1182) using N_2_ as a sole source of nitrogen; *B*, time course of *in vivo* nitrogenase activity determined by whole-cell acetylene reduction assays as described under “Experimental procedures.” Cells were shifted from growth using 10 mm urea as the nitrogen source to media containing no fixed nitrogen at time 0. Activities shown on the *y axis* of *B* indicate nmol of ethylene·min^−1^·*A*_600_^−1^. *Error bars*, S.D.

To be certain that the strains producing either MoFe protein^HisΔZ^ or MoFe protein^StrΔZ^ are phenotypically the same, their growth upon derepression was compared (Fig. 4*A*). The growth features are the same for both *nifZ*-deletion strains and are identical to the originally reported growth features of an *A. vinelandii nifZ*-deletion strain producing MoFe protein having no affinity tag (19). The important feature to note is that, relative to the WT strain producing MoFe protein^Str^, strains deleted for *nifZ* exhibit a noticeable lag when switched to diazotrophic growth conditions. This aspect is also evidenced by a lag in the appearance of whole-cell nitrogenase activity in strains deleted for *nifZ* relative to the WT (Fig. 4*B*). Such a lag is more compatible with slow and undifferentiated maturation of both P-clusters (equivalent function model; Fig. 2*B*) rather than the stepwise function model (Fig. 2*A*) (8, 16–18). The model shown in Fig. 2*B* predicts that upon derepression, a mixed species of MoFe protein captured at various stages in maturation will slowly accumulate, including fully mature MoFe protein, and that the relative amounts of the various species could change over time. In contrast, the stepwise function model shown in Fig. 2*A* predicts that only a half-active MoFe protein for which one half contains FeMo-cofactor and mature P-cluster and the other half contains immature P-cluster and no FeMo-cofactor will accumulate in a NifZ-deficient strain. Although there is an extensive body of literature to support the involvement of NifZ in the stepwise function model shown in Fig. 2*A* (8, 16–18, 22, 25), all of those studies critically rely on the homogeneous accumulation of a 50/50 active and inactive MoFe protein heterotetramer in a strain defective in NifZ, which has been claimed to be experimentally established (16).

### Multiple forms of MoFe protein accumulate in a NifZ-deficient strain

**Figure 5.**
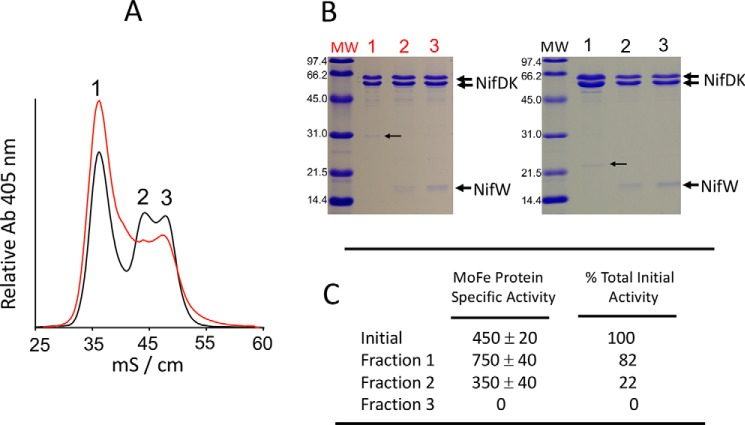
**Separation of nitrogenase species accumulated in NifZ-deficient cells.** *A*, anion-exchange chromatographic profile of MoFe protein^HisΔZ^ previously isolated using IMAC (*red trace*) and ion-exchange chromatography of MoFe protein^StrΔZ^ previously isolated using STAC (*black trace*). The *x axis label* indicates conductivity (mS/cm) as a relative measure of the NaCl concentration in the elution gradient. *B*, SDS-PAGE of peaks from anion-exchange separation of nitrogenase species from MoFe protein^HisΔZ^ (*left*) and MoFe protein^StrΔZ^ (*right*). Protein samples shown here and in other figures were separated using a 4% acrylamide stacking gel and 15% acrylamide running gel and then stained with Coomassie Brilliant Blue. Protein standards in the *left lane* of each gel include phosphorylase B (97.4 kDa), BSA (66.2 kDa), ovalbumin (45.0 kDa), carbonic anhydrase (31.0 kDa), soybean trypsin inhibitor (21.5 kDa), and lysozyme (14.4 kDa). The molar ratios of NifW relative to the MoFe protein α_2_β_2_ heterotetramer in *lanes 2* and *3* (*right*) were estimated to be ∼1 and 2, respectively, based on densitometry. The *small arrow* on the *left* (*lane 1*) indicates enoyl-CoA hydratase, and the *small arrow* on the *right* (*lane 1*) indicates acetyl-CoA carboxylase α-subunit, common contaminants in IMAC and STAC purifications, respectively. The identity of all proteins indicated in the gels was determined by MS as described under “Experimental procedures.” *C*, acetylene reduction specific activities (nmol of ethylene produced·min^−1^·mg^−1^) of MoFe protein species contained in the initial STAC-purified sample and fractions 1, 2, and 3 from anion-exchange chromatography. The percentage of initial activity was calculated based on the total activity present in the initial STAC-purified sample loaded on the anion-exchange column and the total activity recovered in each fraction. *MW*, molecular weight.

In the present work, we tested whether MoFe protein produced in NifZ-deficient *A. vinelandii* is homogeneous (16) or, instead, accumulates as a heterogeneous population of different MoFe protein species. In our first analysis, MoFe protein^HisΔZ^ present in cells derepressed for nitrogen fixation for 4 h was isolated using immobilized metal-affinity chromatography (IMAC)^3^ and subsequently passed over an anion-exchange chromatography column. In contrast to conclusions from the previous report (16), three fractions containing MoFe protein could be identified by this method (*red trace* in Fig. 5*A*). Denaturing gel electrophoresis further revealed that the composition of the three fractions is different (Fig. 5*B*). Fraction 1 contains only MoFe protein subunits, whereas fraction 2 contains MoFe protein subunits and the accessory protein NifW at a molar ratio, based on densitometry, of ∼1.0 relative to the MoFe protein α_2_β_2_ heterotetramer (α_2_β_2_W_1_). Fraction 3 contains MoFe protein subunits and, based on densitometry, twice the amount of NifW found in fraction 2 (α_2_β_2_W_2_; see scheme shown in Fig. 2*B* and the legend to Fig. 5). These same features could be reproduced using Strep-Tactin–based affinity chromatographic purification (STAC) of MoFe protein^StrΔZ^ followed by anion-exchange chromatography (Fig. 5*A*, *black trace*). Because the STAC purification method yielded better resolution of fractions 2 and 3 upon ion-exchange chromatography, the capacity for MoFe protein^StrΔZ^ present in various fractions to support acetylene reduction, a proxy for N_2_ reduction activity, was measured for those samples (Fig. 5*C*). The results of this analysis revealed that the MoFe protein acetylene reduction activity present in the initial sample prepared by the STAC purification method (∼450 nmol·min^−1^·mg^−1^) was only ∼25% of fully active MoFe protein (∼1,800 nmol·min^−1^·mg^−1^ (13, 21), a value much lower than the 50% activity predicted by the stepwise function model. Fraction 1 was enriched for MoFe protein activity relative to the initial sample, whereas fraction 3 had no MoFe protein activity.

**Figure 6.**
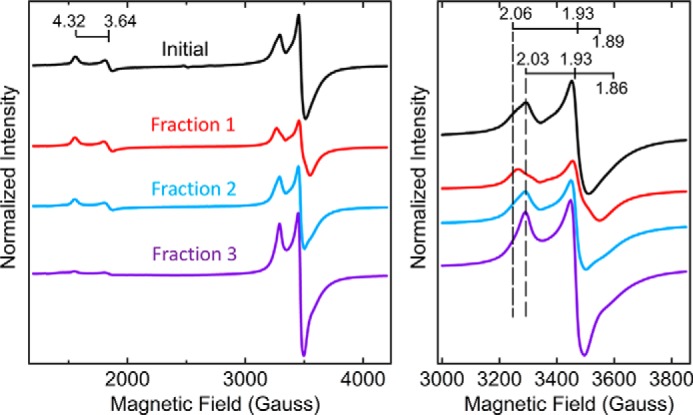
**X-band EPR spectra of MoFe protein^StrΔZ^ isolated by STAC and further fractionated by anion-exchange chromatography.** EPR spectra were acquired from the same samples described in the legend to Fig. 5 and were normalized to a final MoFe protein concentration of 21.7 μm. Fractions 1, 2, and 3 correspond to fractions 1, 2, and 3 shown in Fig. 5*A* (*black trace*). The *right panel* shows an expanded view of the *S* = ½ signature associated with immature P-cluster present in the various samples, which appears split as two electronic isomers (g = 2.03, 1.93, and 1.86 and g = 2.06, 1.93, and 1.89) in all samples except for fraction 3, which appears to only exhibit the g = 2.03, 1.93, and 1.86 species. Note that the g = 2.06, 1.93, and 1.89 species is more dominant in fraction 1 (*red trace*), and the g = 2.03, 1.93, and 1.86 species is more dominant in fraction 2 (*blue trace*).

A comparison of the EPR spectra of the initial MoFe protein^StrΔZ^ sample prepared by the STAC method and fractions 1, 2, and 3 separated by anion-exchange chromatography also revealed differences among them (Fig. 6). Particularly noteworthy is that fraction 3 is enriched for MoFe protein^StrΔZ^ having the *S* = ½ signature assigned to P-cluster precursor, almost no *S* = 32 FeMo-cofactor signature, and no detectable MoFe protein activity. There are also subtle, but reproducible, differences in the line-shape and high-field EPR g-values of the various samples. Namely, the initial sample as well as fractions 1 and 2 appear to have different proportions of two *S* = ½ EPR species, one having g-values of 2.06, 1.93, and 1.89 and the other having g-values of 2.03, 1.93, and 1.86. The appearance of two electronic isomers having the same g-values is also a feature of MoFe protein^StrΔH^ and MoFe protein^StrΔHΔZ^, shown in Fig. 3. In contrast, fraction 3 only exhibits the species having g-values of 2.03, 1.93, and 1.86. Given that fraction 3 is enriched for MoFe protein^StrΔZ^ having a nearly homogeneous *S* = ½ EPR signature and one NifW attached per MoFe protein αβ-unit, it could be that the two apparent EPR species in MoFe protein^StrΔZ^ populations present in other fractions are differentiated by whether or not NifW is, or has been, attached to the MoFe protein. In other words, attachment of NifW or, perhaps, the prior attachment of NifW could alter the protein environment of the P-cluster precursor, resulting in slight changes in its electronic properties compared with samples having not interacted with NifW. This possibility merits further exploration.

**Figure 7.**
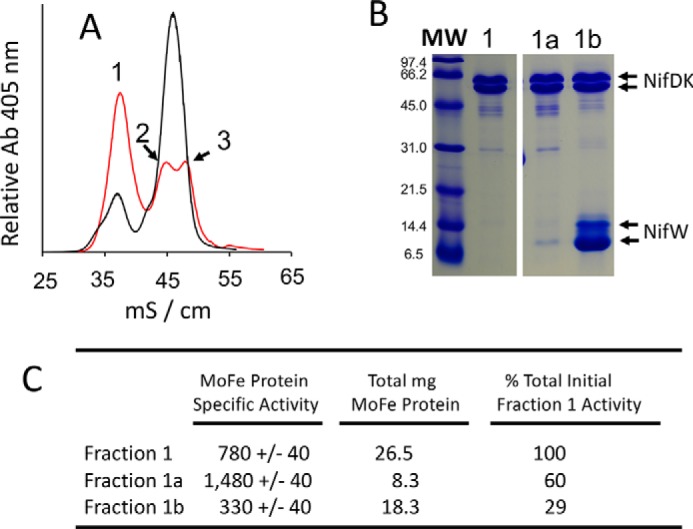
**NifW-assisted fractionation of MoFe protein species produced by NifZ-deficient cells.** *A*, anion ion-exchange chromatographic elution profile of STAC-purified MoFe protein^StrΔZ^ from extracts of DJ2111 cells derepressed for 4 h. For the *black trace*, a 32-fold excess of NifW^Str^ was added to the sample prior to anion-exchange chromatography, and for the *red trace*, no NifW^Str^ was added. The *x* axis indicates conductivity (mS/cm) as a relative measure of the NaCl concentration in the gradient. *B*, SDS-PAGE analysis of NifW^Str^ affinity column separation of different MoFe protein^HisΔZ^ populations. IMAC-purified MoFe protein^HisΔZ^ prepared from cells derepressed for 12 h was further fractionated using anion-exchange chromatography, in the same way as shown in Fig. 5*A* (*red trace*) to obtain fraction 1 (lane 1). Fraction 1 was then passed over a NifW^Str^-charged Strep-Tactin column. Flow-through that is not retained on the column is shown in *lane 1a*. MoFe protein^HisΔZ^ retained on the column and subsequently eluted using biotin to release NifW^Str^ from the Strep-Tactin matrix is shown in *lane 1b*. Note that the band corresponding to NifW in *lane 1a* represents a small amount of NifW^Str^ that has leached off the Strep-Tactin column. Also note that the high level of NifW in *lane 1b* is the result of a large excess of NifW^Str^ bound to the column relative to the amount of MoFe protein^HisΔZ^ applied to the column. Samples shown in *B* were all run on the same SDS-polyacrylamide gel. Other samples also run on the same gel but not relevant to the present work were edited out of the gel picture as indicated by the *space* between *lanes 1* and *1a*/*1b. C*, specific activities (nmol of ethylene produced·min^−1^·mg^−1^) and relative amounts and total activities of MoFe protein^HisΔZ^ present in fractions 1, 1a, and 1b. *MW*, molecular weight.

Because fraction 1 exhibits MoFe protein^StrΔZ^ activity (∼750 nmol·min^−1^·mg^−1^, acetylene reduction assay) that approaches one-half of WT MoFe protein^Str^ activity (∼1,800 nmol·min^−1^·mg^−1^) (13, 21), it seemed possible that this fraction could represent a homogeneous sample for which half of each MoFe protein contains FeMo-cofactor and intact P-cluster and the other half contains immature P-cluster and no FeMo-cofactor, consistent with the stepwise function model. Another possibility is that fraction 1 represents a mixed population that includes fully mature MoFe protein as well as immature MoFe protein species. It was anticipated that these possibilities could be distinguished by asking whether the addition of NifW might shift a portion of MoFe protein^StrΔZ^ within fraction 1 toward fractions 2 and 3 in the anion-exchange chromatography step. The rationale for considering this possibility is that, because MoFe protein is produced at a much higher abundance than the NifW assembly factor (26), the amount of NifW available for interaction with MoFe protein having immature P-clusters *in vivo* must be limited in a strain deleted for *nifZ*. Also, MoFe protein having immature P-clusters but no NifW attached should have the same anion-exchange elution profile as intact MoFe protein, whereas MoFe protein having one or two NifW attached should elute at respectively higher salt concentrations. The latter possibility is consistent with the low pI of NifW (calculated pI ∼4.47) and was experimentally established by data shown in Fig. 5*A*. It was also anticipated that the elution profile of any fully mature MoFe protein present within fraction 1 would not be affected by the addition of NifW because NifW does not interact with the mature MoFe protein. For the experiment shown in Fig. 7*A*, MoFe protein^StrΔZ^ was purified using the STAC protocol and split into two separate samples. One sample was directly fractionated by anion-exchange chromatography (Fig. 7*A*, *red trace*), whereas the other sample was fractionated after the addition of a 32-fold excess of isolated NifW relative to MoFe protein (Fig. 7*A*, *black trace*). The result of this experiment reveals that the addition of purified NifW shifts a portion of fraction 1 to fractions 2 and 3, clearly indicating that fraction 1 is not a homogeneous sample. Measurement of MoFe protein^StrΔZ^ activity remaining in fraction 1 after the addition of NifW and anion-exchange chromatography was ∼1,400 nmol·min^−1^·mg^−1^ (acetylene reduction assay) compared with the activity of fully mature MoFe protein^Str^ (∼1,800 nmol·min^−1^·mg^−1^), indicating that a relatively high portion of the MoFe protein^StrΔZ^ present in the original fraction 1 represents fully mature MoFe protein replete with intact P-clusters and FeMo-cofactor.

**Figure 8.**
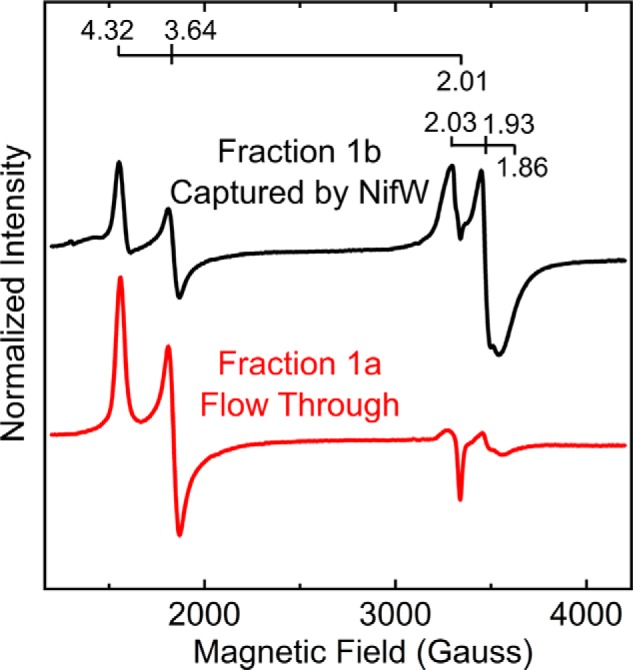
**X-band EPR spectra of MoFe protein^HisΔZ^ populations separated by NifW affinity chromatography.** The *black trace* is the EPR spectrum obtained from fraction 1b shown in Fig. 7*B*, and the *red trace* is the EPR spectrum of fraction 1a, also shown in Fig. 7*B*. Both spectra were normalized to a final MoFe protein concentration of 21.7 μm.

The preceding result was subsequently confirmed in a different way by using a NifW-affinity column to separate MoFe protein^HisΔZ^ that only contains mature P-clusters from those that contain one or two immature P-clusters. In this experiment, fraction 1 from MoFe protein^HisΔZ^ prepared by the IMAC procedure and anion-exchange chromatography (see *red trace* in Fig. 5*A*) was passed over a Strep-Tactin column charged with Strep-tagged NifW (21). According to the results presented in Fig. 7*A*, any population of MoFe protein^HisΔZ^ contained in fraction 1 that does not harbor immature P-clusters is expected to pass through the column, whereas any population containing either one or two immature P-clusters should be retained on the column. The bound fraction, captured by NifW, could be subsequently eluted for analysis by application of a biotin-containing buffer (21). The results of this experiment reveal that passing fraction 1 over the NifW-affinity column, yielding fraction 1a (flow-through), highly enriches MoFe protein activity relative to fraction 1b, which is retained on the column (Fig. 7*C*). Such differential enrichment was also confirmed by comparison of the EPR profiles of MoFe protein^HisΔZ^ contained in fraction 1a and fraction 1b (Fig. 8). It is also noteworthy that the *S* = ½ EPR spectrum of MoFe protein^HisΔZ^ captured by NifW appears to be almost entirely a single species having g-values of 2.03, 1.93, and 1.86, further indicating that populations of P-cluster precursor in MoFe protein^HisΔZ^ having NifW attached could be in slightly different environments from those that do not.

### The complement of mature MoFe protein produced by a strain deleted for nifZ increases over time following derepression

Derepression of *A. vinelandii* for nitrogen fixation results in the immediate expression of the nitrogen-fixation components (27, 28). According to the stepwise function model, only MoFe protein that is 50% active, having one αβ-unit containing one FeMo-cofactor and one mature P-cluster and the other αβ-unit having one immature P-cluster and no FeMo-cofactor, should accumulate in NifZ-deficient strains during the derepression process. In contrast, the equivalent function model predicts that the relative population of mature and immature MoFe protein could change over time in NifZ-deficient strains following derepression, including a time-dependent enrichment of fully mature MoFe protein. These possibilities were explored by comparing the relative activities of IMAC-purified MoFe protein^HisΔZ^ and STAC-purified MoFe protein^StrΔZ^ prepared from cells that had been derepressed for 4 or 12 h. Indeed, the total relative MoFe protein activities in these samples change over time, exhibiting an approximate increase in 50% for cells harvested after 12 h derepression compared with those harvested after 4 h derepression (Table 2).

**Table 2:** **Specific activities of MoFe protein isolated from NifZ-deficient cells that were derepressed for nitrogenase expression for either 4 or 12 h**

Source/Genotype	Purification	Derepression	Activity
		*h*	*nmol ethylene produced*·*min*^−*1*^·*mg*^−*1*^ *MoFe protein*
DJ1182/MoFe protein^HisΔZ^	IMAC	4	400 ± 30
DJ1182/MoFe protein^HisΔZ^	IMAC	12	640 ± 30
DJ2111/MoFe protein^StrΔZ^	STAC	4	450 ± 20
DJ2111/MoFe protein^StrΔZ^	STAC	12	640 ± 30

### Further evidence that NifZ is not required for maturation of either P-cluster

**Figure 9.**
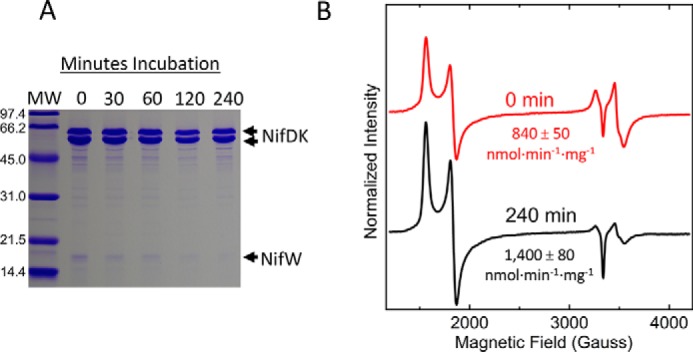
**Time-dependent *in vitro* maturation of ΔnifZ nitrogenase in cell-free extracts.** MoFe protein^StrΔZ^ cell-free extract prepared from DJ2111 cells derepressed for 12 h was incubated at different times (0, 30, 60, 120, and 240 min) in the presence of 36 mm Na_2_S_2_O_4_. *A*, SDS-PAGE of MoFe protein^StrΔZ^ purified by STAC after 0-, 30-, 60-, 120-, and 240-min incubation. *B*, X-band EPR spectra of MoFe protein^StrΔZ^ after processing the 0-min sample and the 240-min sample by anion-exchange chromatography and selecting the first elution peak (fraction 1). Both spectra were normalized to a final MoFe protein concentration of 21.7 μm. The corresponding MoFe protein^StrΔZ^ acetylene reduction specific activities (nmol of ethylene produced·min^−1^·mg^−1^) of those samples are indicated *below* the spectra. *MW*, molecular weight.

An aspect that distinguishes the stepwise function model for NifZ involvement in P-cluster maturation from the equivalent function model is that the former demands that NifZ be required for maturation of the so-called “second” P-cluster (8, 16), whereas the latter does not. The dispensability of NifZ for full formation of mature MoFe protein, established by experiments already described, was also shown by another method. In this latter case, crude extracts prepared from cells derepressed for MoFe protein^StrΔZ^ for 12 h were simply incubated over a time course in the presence of excess reducing agent, dithionite, without the addition of any other proteins or factors. MoFe protein^StrΔZ^ was subsequently purified from each time point using STAC and analyzed by PAGE. The results of these experiments reveal the slow, time-dependent and NifZ-independent, dissociation of NifW (Fig. 9*A*). Anion-exchange chromatography was used to further process the initial sample and the sample incubated for 4 h. Analysis of fraction 1 from these samples revealed an approximate doubling in MoFe protein activity (Fig. 9*B*), a relative increase in the *S* = 32 EPR signature associated with FeMo-cofactor, and a marked decrease in the *S* = ½ EPR signature associated with immature P-cluster of MoFe protein^StrΔZ^ as a result of the 4 h incubation (Fig. 9*B*). See the legend and *black trace* in Fig. 5*A* for an example of preparation of fraction 1 by this method. Most notably, the activity achieved following a 4 h incubation is ∼78% of the theoretical value of fully active MoFe protein^Str^ (Fig. 9*B*), indicating that maturation of both P-clusters occurs in the absence of NifZ. In contrast to the situation with extracts prepared from a NifZ-deficient strain, which contain NifH (Fe protein), extracts prepared from a strain deficient in Fe protein exhibited no MoFe protein activity, and no activation could be achieved by prolonged incubation of those extracts. It has been shown previously that activation of MoFe protein contained in extracts of an Fe protein-deficient strain requires the addition of both ATP and Fe protein (12).

## Discussion

**Figure 10.**
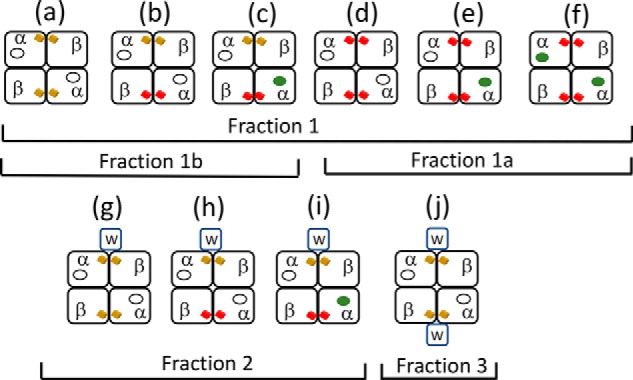
***Schematic representation* of theoretical MoFe protein species that could accumulate in ΔnifZ mutant cells.** Fractions indicated correspond to those shown in Figs. 5 and 7.

Fig. 10 schematically shows MoFe protein species that could theoretically accumulate in NifZ-deficient cells if NifZ has an equivalent role in the maturation of both immature P-cluster species. It also provides a framework for an interpretation of results obtained in the present work. Because FeMo-cofactor is expected to be rapidly inserted after P-cluster maturation (29, 30), it is unlikely that species *b*, *d*, *e*, or *h* shown in Fig. 10 accumulate to significant levels in NifZ-deficient extracts. Purification of MoFe protein^HisΔZ^ or MoFe protein^StrΔZ^ by IMAC or STAC, respectively, captures all of the different MoFe protein species that could accumulate in the corresponding samples. We have shown that samples prepared by either IMAC or STAC can be further processed by anion-exchange chromatography to yield three fractions that are differentiated by their respective activities, EPR spectra, and whether or not NifW is attached. The species contained in fraction 3, which has a robust *S* = ½ EPR signature, almost no *S* = 32 EPR signature, two NifW attached, and no activity, is likely to represent species *j* in Fig. 10. The species contained in fraction 2 has both *S* = 32 and *S* = ½ EPR signatures, exhibits an intermediate level of activity, and probably has only one NifW attached. Thus, only species *g*, *h*, and *i* can be present in fraction 2. Because the separately formed FeMo-cofactor is expected to be rapidly inserted once P-cluster maturation is complete, as noted above, it is probable that *g* and *i* are the dominant species in fraction 2. The composition of fraction 1 is more complicated because there are six different species that could theoretically populate that fraction. However, fraction 1 could be further processed using NifW-affinity chromatography to enrich for MoFe protein^HisΔZ^ that either contains (fraction 1b) or does not contain (fraction 1a) immature P-clusters. Given that the specific activity of MoFe protein^HisΔZ^ present in fraction 1a represents ∼82% of that of fully active MoFe^His^ protein, species *f*, mature MoFe protein, must be the dominant form present in fraction 1a. Because at least some of the MoFe protein^HisΔZ^ present in fraction 1b exhibits both the *S* = 32 signal assigned to FeMo-cofactor and the *S* = ½ signal assigned to immature P-cluster, species *c* must be present in fraction 1b. In aggregate, these findings, together with the observations that the complement of mature MoFe protein produced by a strain deleted for *nifZ* increases over time following derepression and that NifZ is not essential for maturation of either P-cluster, are only compatible with the equivalent function model shown in Fig. 2*B*. This model is also in line with the observations that P-cluster formation/maturation involves the sequential and differential interaction of immature MoFe protein with at least three assembly factors, NafH, NifW, and NifZ, that are not absolutely essential for P-cluster formation/maturation (13, 19), as well as NifH (Fe protein), which is essential for P-cluster maturation (8).

The exact function of the nonessential assembly factors associated with P-cluster formation/maturation is not known, but the present work, as well as previous reports (8, 13, 16, 26, 31, 32), provides a basis for informed speculation and is summarized in Fig. 2*B*. It is known that an *A. vinelandii* strain deleted for *nifW* accumulates fully active MoFe protein at lower abundance than WT (19, 31, 32), NifZ functions after NifW (13), both NifW and NifZ sequentially interact with immature MoFe protein (13), and NifH is essential for converting immature P-clusters to the mature form (8). These observations lead us to speculate that NifW plays an important but nonessential role in stabilizing a form of MoFe protein that is primed to receive Fe/S species during an early step in the assembly of immature P-clusters and that, in the absence of immature P-cluster formation, the MoFe protein subunits are degraded. Once immature P-clusters are formed, NifZ might play a role in the dissociation of NifW and, perhaps, aid in recruiting Fe protein to complete nucleotide- and reductant-dependent P-cluster maturation. In summary, results reported here do not support the proposal that NifZ is involved in stepwise P-cluster maturation but, instead, indicate that NifZ performs a nonessential and equivalent role in the maturation of both P-clusters.

## Experimental procedures

### Strains and plasmids

Strains used in this study are listed in Table 1. Defined deletions were incorporated into the *A. vinelandii* genome by congression or marker rescue using transformation of competent cells using recombinant plasmid DNA having defined deletions (13, 33).

### Growth conditions

*A. vinelandii* cells were grown at 30 °C in a 150-liter custom-built fermenter (W. B. Moore, Inc., Easton, PA) in modified Burk medium (34) containing 10 μm Na_2_MoO_4_ as the Mo source and 10 mm urea as a nitrogen source. Parameters for growth and cell harvesting were the same as described previously (20, 21), and derepression of nitrogenase formation was for either 4 or 12 h as indicated in Table 2 and the figure legends. *Escherichia coli* strain BL21 (DE3) was used as the host for plasmid pDB2108, which directs the expression of Strep-tagged NifW (13, 21).

### Purification of Strep-tagged MoFe protein from A. vinelandii, Strep-tagged NifW from recombinant E. coli, and affinity purification using Strep-tagged NifW as bait

All purification of Strep- or His-tagged MoFe proteins was carried out as described in detail previously (21), using either Strep-Tactin (IBA Lifesciences, Göttingen, Germany) or immobilized metal affinity (GE Healthcare) columns. When anion-exchange chromatography was used for further purification, ∼20 mg of MoFe protein isolated using either the STAC or IMAC procedure was applied to and eluted from a 1-ml Q-Sepharose HP column (GE Healthcare), using a 200–500 mm NaCl gradient, 20-ml total volume.

### In vitro maturation of NifZ in a NifZ-free assay

Cell-free extract from 150 g of DJ2111 cells was prepared in 50 mm Tris-HCl, pH 7.9, 1.6 mm Na_2_S_2_O_4_ as described previously (21), aliquoted into five separate 100-ml serum bottles, and maintained under an Ar atmosphere. Na_2_S_2_O_4_ was added to each sample to give a final concentration of 36 mm and incubated with gentle agitation at 30 °C (35). Samples incubated for 0, 30, 60 120, and 240 min were then individually applied to a STAC column for isolation of MoFe^StrΔZ^. Preparation of samples for nitrogenase activity assays and EPR was as described above using anion-exchange chromatography.

### Acetylene reduction assays

Ethylene gas formed in nitrogenase activity assays was detected using a flame ionization detector coupled to a Shimadzu GC-2010-plus gas chromatograph equipped with a Carboxen® 1010 plot column. Specific activity is defined as nmol of product formed per min per mg of Strep-tagged MoFe protein. *In vivo* nitrogenase activity units are defined as nmol of ethylene formed per min per ml of culture at an *A*_600_ equal to 1. *In vitro* assays were performed using 9-ml serum vials sealed with serum stoppers under an Ar atmosphere. Reactions were initiated by injection of 0.8 ml of acetylene. All 1-ml assay mixtures included 15 μg of MoFe protein, 165 μg of Fe protein, 8 mm Na_2_S_2_O_4_, 50 μg/ml creatine phosphokinase, 8 mm MgCl_2_, 1.35 mm ATP, and 18 mm phosphocreatine. Fe protein used for nitrogenase assays was prepared from strain DJ0033 (Table 1) deleted for the *nifD*- and *nifK*-encoding subunits to ensure that no substrate reduction activities could be attributed to contaminating MoFe protein present in Fe protein samples used for assays. Fe protein was purified as described previously (36). Assay samples were incubated at 30 °C with agitation for 15 min and were terminated by the injection of 100 μl of 4 n NaOH. To determine *in vivo* acetylene reduction activity of *A. vinelandii* strains, 1 ml of each cultured strain was transferred to 9-ml sealed vials with a 92% air, 8% acetylene gas phase and incubated at 30 °C for 15 min and were terminated by the injection of 100 μl of 4 n NaOH. All acetylene reduction activities were performed in triplicate.

### Protein determination by BCA and densitometry

Based on total protein concentrations determined by the BCA method (37), ImageJ software was used to plot SDS-polyacrylamide gel lanes from high-quality images to calculate the concentration of MoFe protein and NifW-specific protein in various samples (38).

### EPR spectroscopy

Continuous-wave X-band EPR spectra were recorded using a Bruker ESP-300 spectrometer with an EMX PremiumX microwave bridge and an EMX^PLUS^ standard resonator in perpendicular mode, equipped with an Oxford Instruments ESR900 continuous helium flow cryostat using VC40 flow controller for helium gas. Spectra were recorded in 4-mm calibrated quartz EPR tubes (Wilmad LabGlass, Vineland, NJ) at the following conditions: temperature, ∼12 K; microwave frequency, ∼9.38 GHz; microwave power, 20 milliwatts; modulation frequency, 100 kHz; modulation amplitude, 8.14 G; time constant, 20.48 ms. Each spectrum represents the sum of 5 or 10 scans. The cavity background signal was recorded using an EPR tube filled with 100 mm MOPS buffer at pH 7.3 and was subtracted from the experimental spectra. For comparison, spectra presented in each figure were normalized to the same concentration of MoFe protein as indicated in the figure legends.

### Protein identification from SDS-PAGE bands via MALDI analysis

All proteins referred to in the present work were identified using MS. Gel bands were excised from SDS-polyacrylamide gels and destained using a 1:1 mixture of 50 mm ammonium bicarbonate (AmBic)/LCMS-grade acetonitrile. Destained gel pieces were dehydrated using LCMS-grade acetonitrile and treated sequentially with 10 mm DTT in 50 mm AmBic for 1 h at 37 °C, 50 mm iodoacetamide in 50 mm AmBic for 30 min at room temperature in the dark, and 100 mm DTT in 50 mm AmBic to quench unreacted iodoacetamide. After washing the gel pieces again with the destaining solution and dehydrating them using LCMS-grade acetonitrile, sufficient 10 ng/μl trypsin in 50 mm AmBic was added to cover the gel pieces, and the samples were incubated overnight at 37 °C.

The following day, 80:20 LCMS-grade acetonitrile/LCMS-grade water supplemented with 0.1% (v/v) formic acid was added at the same volume as the trypsin solution used the previous day. Samples were incubated in a sonicating water bath for 10 min, and then 1 μl of each sample was spotted onto a MALDI target plate. Once the sample dried, 1 μl of matrix solution (2 mg/ml α-cyano-4-hydroxycinnamic acid in 50:50 LCMS-grade acetonitrile/LCMS-grade water supplemented with 0.1% (v/v) TFA was added. After drying, samples were analyzed using a 4800 MALDI-Tof/Tof (AB Sciex) first in positive-ion reflector mode to obtain peptide masses then in positive-ion MSMS1kV mode to obtain tandem MS (MS2) spectra of the 12 most intense peaks observed in the MS1.

The resultant peak lists were searched against the combined Uniprot and NCBInr protein databases using the Mascot (Matrix Science) web server. Search parameters used were trypsin specificity with the possibility of one missed cleavage, a precursor tolerance of 250 ppm, a product tolerance of 0.25 Da, carbamidomethylation of cysteine residues as a fixed modification, and oxidation of methionine and pyroglutamate formation of glutamine residues found at the N terminus of a peptide as variable modifications. Proteins were confidently identified when at least three unique peptides were found or if at least one peptide with a Mascot score above 50 was identified and manually validated. In the case of NifW, two peptides (AGDLDEHDDQAR and AYLDFVESDALTEK), having a Mascot score of 94, were identified.

## Author contributions

E. J.-V. and D. R. D. conceptualization; E. J.-V. data curation; E. J.-V., Z.-Y. Y., and D. R. D. formal analysis; E. J.-V., J. S. M. D. C., and V. L. C. investigation; E. J.-V. and Z.-Y. Y. methodology; E. J.-V. and D. R. D. writing-original draft; E. J.-V., Z.-Y. Y., J. S. M. D. C., V. L. C., and L. C. S. writing-review and editing; L. C. S. and D. R. D. funding acquisition; L. C. S. and D. R. D. project administration.

## Footnotes

3 The abbreviations used are:

IMAC: immobilized metal-affinity chromatography
STAC: Strep-Tactin–based affinity chromatographic purification
AmBic: ammonium bicarbonate.
